# Microbial efficiency enhancement drives carbon sequestration in long-term organic farming systems: linking taxonomic succession to carbon use efficiency

**DOI:** 10.3389/fmicb.2026.1770908

**Published:** 2026-03-16

**Authors:** Bo Ram Kang, Young Jun Bae, Soundarya Rajapitamahuni, Min-Seob Kim, Soon-Jae Lee, Youngmi Lee, Hong Shik Nam, Tae Kwon Lee

**Affiliations:** 1 Department of Environmental and Energy Engineering, Yonsei University, Wonju, Republic of Korea; 2 Climate Change and Carbon Research Department, Environmental Standards Research Division, National Institute of Environmental Research, Incheon, Republic of Korea; 3 Department of Ecology and Evolution, University of Lausanne, Lausanne, Switzerland; 4 African Genome Center and University Mohammed VI Polytechnic (UM6P), Ben Guerir, Morocco; 5 Organic Agriculture Division, National Institute of Agricultural Sciences, Wanju, Republic of Korea

**Keywords:** carbon stabilization mechanisms, carbon use efficiency, microbial succession, Mortierellomycetes, organic farming

## Abstract

Organic farming enhances soil carbon sequestration, which is a critical strategy for climate change mitigation and sustainable agriculture. However, the microbial mechanisms driving carbon accumulation in the soil, particularly the role of metabolic efficiency in long-term organic systems, remain poorly understood. We investigated microbial succession, metabolic efficiency, and carbon stabilization across an organic farming chronosequence (0–5, 5–10, and >10 years) in pepper and cabbage systems. We measured soil carbon fractions, glomalin-related soil proteins, microbial community composition, carbon use efficiency, and extracellular enzyme activities. Organic management beyond a critical 10-year threshold enhanced soil organic matter by 108% and total glomalin-related soil proteins by 4.0-fold compared with conventional farming, with no significant accumulation during the initial 5 years. This non-linear pattern corresponded with a 3.7-fold enhancement in the microbial carbon use efficiency (CUE) measured via dual-isotope approaches (^13^C-glucose and ^18^O-H₂O). Taxonomically coherent succession revealed a positive correlation between Mortierellomycetes proliferation and CUE (rho = 0.67–0.71), whereas inefficient Gammaproteobacteria declined. The eco-enzymatic stoichiometry shifted from 81.7 to 10.1 indicating reduced nitrogen and phosphorus limitation and enhanced carbon acquisition. Correlation network analysis identified CUE as the master regulator linking microbial community structure to carbon stabilization. Our findings establish metabolic efficiency enhancement, rather than biomass accumulation, as the primary mechanism driving soil carbon sequestration under organic management, providing actionable biomarkers for monitoring transition progress and optimizing carbon-smart agricultural practices.

## 1 Introduction

Soil organic carbon, comprising 1,500–2,400 Pg C globally, represents the largest terrestrial carbon reservoir and exceeds atmospheric carbon by 2–3-fold (Bonfanti et al., 2025; Jobbágy and Jackson, 2000). The “4 per mille” initiative highlights that increasing soil carbon stocks by only 0.4% annually could offset anthropogenic CO₂ emissions, positioning agricultural soils as critical climate mitigation tools (Bruni et al., 2024; Koutika et al., 2025). Organic farming practices eliminate synthetic inputs and emphasize biological processes, which have been promoted as strategies to enhance soil carbon sequestration (Koorneef et al., 2025). However, sequestration outcomes vary dramatically across studies, with some reporting minimal gains after decades of organic management while others document substantial increases within years (Beillouin et al., 2023). Recent meta-analyses have revealed a critical pattern in which significant carbon accumulation often requires more than 10 years of continuous organic practice (Beillouin et al., 2023; Meng et al., 2024); however, the biological mechanisms underlying these temporal thresholds remain poorly understood. Without mechanistic insights into these time-dependent processes, we cannot predict when organic transitions will succeed in climate change mitigation or in designing policies that sustain practices through initial low-return periods.

Traditional soil carbon models operate under a biomass-centric paradigm, assuming that increased microbial biomass drives enhanced carbon storage (Mason-Jones et al., 2023). However, this assumption fails to explain why systems with high microbial biomass often show poor carbon sequestration; in contrast, those with modest biomass accumulate substantial carbon (Mason-Jones et al., 2023). Carbon use efficiency (CUE), the proportion of assimilated carbon allocated to growth versus respiration, has emerged as a critical missing link between microbial community structure and ecosystem-level carbon dynamics (Tao et al., 2023). Recent theoretical frameworks suggest that organic farming transitions trigger a fundamental metabolic shift in soil microbial communities (Liu et al., 2024; Ma et al., 2023). This shift proceeds from communities dominated by copiotrophic bacteria, which exhibit low CUE and rapid turnover, to oligotrophic fungi, which are characterized by high CUE and slow growth. Emerging evidence indicates that specific fungal taxa, particularly Mortierellomycetes, possess inherently superior carbon conversion efficiencies compared to bacterial groups (Wu et al., 2025). However, no study has directly linked specific microbial taxa to CUE measurements during organic farming transitions, leaving the metabolic efficiency filter hypothesis untested and preventing the development of efficient management strategies.

Soil enzymatic stoichiometry provides a window into the microbial metabolic strategies and resource allocation patterns that govern carbon cycling (Quan et al., 2025). The ratio of nitrogen- and phosphorus-acquiring enzymes to carbon-acquiring enzymes reflects microbial nutrient demands and reveals whether communities are carbon- or nutrient-limited (Wan et al., 2025). Recent studies have shown that the alleviation of nutrient limitation through organic amendments should reduce investment in nutrient-acquiring enzymes, allowing greater allocation to carbon processing (Balami et al., 2025; Gurmessa et al., 2024). Concurrently, glomalin-related soil proteins (GRSP) may represent a physical manifestation of enhanced metabolic efficiency (Singh et al., 2024; Wang et al., 2024). These recalcitrant compounds contribute 20–30% of soil organic carbon in some systems and play crucial roles in aggregate formation (Driver et al., 2005). However, their production dynamics during organic transitions remain unexplored. Furthermore, crop identity modulates root exudation patterns and mycorrhizal associations, potentially creating distinct carbon sequestration pathways (Camli-Saunders and Villouta, 2025; Sharma et al., 2023). However, comparative studies examining crop-specific effects on the complete microbe-enzyme-carbon pathway are lacking.

We hypothesized that long-term organic farming creates a metabolic efficiency filter that progressively selects high CUE taxa and fundamentally reorganizes soil carbon cycling through interconnected biological and biochemical mechanisms. Using a chronosequence approach spanning 0–5, 5–10, and > 10 years of organic management across two contrasting crop systems (pepper and cabbage), we aimed to (i) quantify temporal thresholds for soil carbon and GRSP accumulation identifying when significant sequestration begins, (ii) characterize microbial community succession using 16S rRNA and 18S rRNA amplicon sequencing to identify indicator taxa, (iii) measure microbial CUE using dual-isotope methods (^13^C-glucose and ^18^O-H₂O) and directly correlate efficiency with specific taxa, and (iv) analyze shifts in enzymatic activities and stoichiometry to understand metabolic reorganization. By mechanistically linking microbial populations to metabolic functions and carbon stabilization, this study provides the first empirical validation of the efficiency-centered paradigm in agricultural systems. Our findings provide CUE-based biomarkers for the rapid assessment of soil health transitions and reveal why temporal persistence drives climate benefits in organic agriculture.

## 2 Materials and methods

### 2.1 Study area and soil sample collection

Soil was sampled in October 2022 in Goesan, South Korea. The target crops were chili peppers (*Capsicum annuum* var. *annuum*) and Chinese cabbage (*Brassica rapa subsp. pekinensis*), which are commonly grown in Korea. Ten fields were investigated from two crops with a 0–5 yrs. organic farming period: Org A, 5–10 yrs.; Org B, > 10 yrs.; Org C; and two fields of conventional farming (Con). In each field, rhizosphere soil samples were collected from three points spaced at least 10 m apart. All organic fields followed standardized crop-specific management protocols maintained consistently over the past decade. Pepper fields employed a 2–3 year rotation cycle with maize and legumes, combined with winter rye cover cropping for green manure. Nutrient management included continuous side-dressing applications throughout the extended growing season from May to October to support fruit development. Cabbage fields utilized a double-cropping system where spring potato or maize was followed by fall cabbage with systematic recycling of crop residues as organic amendments. Foliar applications of chlorella culture medium and microbial inoculants were used to enhance tissue quality. Conventional fields employed heterogeneous management practices typical of non-certified farming systems in the region. Based on farmer surveys, conventional practices generally included NPK compound fertilizers (200–250 kg N ha^−1^ yr.^−1^), and synthetic pesticides (fungicides and insecticides applied at 7–14 day intervals). Specific application rates and timing varied among individual farmers. For each cropping system, three independent field replicates (*n* = 3) were sampled for each organic farming treatment (Org A, Org B, and Org C), which followed consistent organic management protocols. Six field replicates (*n* = 6) were sampled for the conventional control to account for the greater management variability inherent in conventional farming practices. At each point, three random soil points within a 10 cm radius were collected from the ridge (10 cm radius and 15 cm depth; approximately 300 g for each sample), with the top 5 cm of the soil layer removed to avoid exogenous disturbance. Thirty soil samples were collected from roots in sterilized polyvinyl chloride bags and transported immediately to the laboratory in an icebox. Raw soil samples were sieved through a 2-mm-diameter mesh and stored at 4 °C until use. Soil subsamples for molecular analysis were stored in a freezer at −80 °C until use.

### 2.2 Soil physicochemical analyses

The soil texture was measured using the pipette method (Mwendwa, 2022). Soil aggregate distribution was analyzed by wet sieving using a Yoder apparatus (Yoder, 1936). Soil aggregates were divided into micro (< 250 μm) and macro (250–2,000 μm) size fractions according to the methodology of Schweizer et al. (2019). Soil moisture content was measured by weight loss to constant weight after oven drying at 105 °C. The water holding capacity (WHC) was estimated using the gravimetric water content.

Soil pH was determined in a 1:5 soil/water suspension using a portable multiparameter meter (Orion Star™ A329, Thermo Scientific, MA, United States). Total carbon (TC) and total nitrogen (TN) were analyzed using an elemental analyzer (Vario MICRO Cube, Elementar, Langenselbold, Hesse, Germany). Soil organic matter (SOM) content was determined using the K_2_Cr_2_O_7_-H_2_SO_4_ oxidation–reduction colorimetric method (Walkley and Black, 1934). NH_4_-N and NO_3_-N were determined via the Kjeldahl method using an automated distillation analyzer (Kjeltec 8,200, Foss, Hillerød, Hovedstaden, Denmark). Available P was extracted using the Lancaster method and detected at 720 nm using a UV/Vis spectrophotometer (Libra S80; Biochrom, Cambridge, United Kingdom). Exchangeable potassium, calcium, magnesium, and sodium levels were estimated using an inductively coupled plasma optical emission spectrometer (iCAP PRO XP DUO; Thermo Scientific, Waltham, MA, United States) after extraction with 1 M NH_4_OAc. The cation exchange capacity (CEC) was evaluated by adding the exchangeable cation results.

### 2.3 Glomalin-related soil proteins

Extraction of GRSP was based on Wright and Upadhyaya (1998). Easily extractable GRSP (EG) was extracted by autoclaving (121 °C) 1 g of soil in 8 mL of 20 mM sodium citrate adjusted to a pH of 7 for 30 min. Total GRSP (TG) was extracted by autoclaving (121 °C) 1 g of soil in 8 mL of 50 mM sodium citrate buffer for 90 min. The extraction was repeated until the supernatant was almost clear. The samples were centrifuged at 5,000 × g for 15 min immediately after extraction; the protein extracted from the supernatant was quantified using a Bradford protein assay kit (Bio-Rad, Hercules, CA, United States).

### 2.4 Soil enzymatic activity

Soil enzyme activity was determined by measuring the absorbance of colorimetric substrates using a microplate reader (Spark 10 M, Tecan, Switzerland). Dehydrogenase activity (DHA) was detected according to Casida et al. (1964) using triphenyltetrazolium chloride (TTC) as a substrate. The formed triphenylformazan was measured at 485 nm and expressed as μg TPF g^−1^ dry soil. *β*-glucosidase (BG), N-Acetyl-β-glucosaminidase (NAG), and acid phosphatase (PP) were measured following Stott et al. (2010) using p-nitrophenol (pNP) as a substrate. Production of p-nitrophenol, resulting from the enzymatic reaction, was measured at 405 nm. Enzymatic activities were expressed as μg p-NP g^−1^ of dry soil.

### 2.5 Carbon use efficiency

The ^13^C-CUE and ^18^O-CUE methods were used to evaluate the soil microbial carbon use efficiency according to Geyer et al. (2019). Soil samples were pre-incubated in aluminum cups for 48 h at 15 °C before measurement.

#### 2.5.1 ^13^C-CUE method

Four replicates of each soil (30 g each) received a glucose amendment (0.05 mg glucose-C g^−1^ dry soil) prepared with universally labeled 99 at% ^13^C-glucose to achieve total glucose enrichment of 5 at%. One control soil sample was treated with a non-labeled glucose solution. After mixing with a spatula, the soils were transferred to 500 mL incubation jars. The jars were sealed with rubber septa, flushed for 15 min with CO_2_-free air, and incubated for 24 h at 15 °C. Before harvesting, 15 mL of headspace was sampled using a syringe and injected into the evacuated exetainers. The CO_2_ concentration was determined using Gas Chromatography–Mass Spectrometry (GC–MS) (GCMS-QP2020, Shimadzu, Kyoto, Japan). The *δ*^13^C of the CO_2_ was measured using an isotope ratio mass spectrometer with trace gas analyzer equipment (TG-IRMS; Trace gas–Isoprime 100; Isoprime Ltd., Manchester, United Kingdom). The isotopic data were reported using delta notation with ^13^C/^12^C variations relative to the international standard Vienna Pee Dee Belemnite (V-PDB):


$$\delta \;(‰)=[(R_{\text{sample}})/(R_{\text{standard}})–1\;]\times 1,000$$

Where *R* = (*^13^C/^12^C*). The analytical precision was within 0.3‰ for CO_2_ gas. The soils were immediately extracted for microbial biomass analysis as described in Section 2.4. The total C in the K_2_SO_4_ extracts was measured using a TOC analyzer (TOC-L; Shimadzu, Kyoto, Japan). The K_2_SO_4_ extracts (20 mL) were freeze-dried and the δ^13^C of the extracts was analyzed using a Gasbench equipped with a 50 μL loop and a PoraPLOT Q GC-Column connected to a Delta-V advantage IRMS (Thermo Scientific, Waltham, MA, United States). CUE was calculated as the ratio of carbon incorporated into microbial biomass to total carbon uptake by Geyer et al. (2019).


$$\mathrm{CUE}=(\Delta \text{Cmic}\times \text{Emic})/(\Delta \text{Cmic}\times \text{Emic}+{\mathrm{CO}}_{₂}-C\times {\mathrm{ECO}}_{₂})$$

Where ΔCmic is the change in microbial biomass carbon, Emic is the 13C enrichment of microbial biomass, CO₂-C is the cumulative respired carbon, and ECO₂ is the 13C enrichment of respired CO₂.

#### 2.5.2 ^18^O-CUE method

Two replicates of each soil (200 mg) were weighed in 1.5 mL plastic vials and inserted into 20 mL glass headspace vials. Soils were labeled with ^18^O–H^2^O (97.0 at%, Sigma Aldrich, St. Louis, MO, United States) to achieve 20.0 at% for ^18^O in the final soil water and 60% WHC. One control group received the same volume of non-labeled deionized water. After mixing by spatula, vials were sealed with rubber septa, flushed with CO_2_-free air, and incubated for 24 h at 15 °C. Before harvesting, 15 mL of headspace was sampled using a syringe and injected into the evacuated exetainers. The CO_2_ concentration was determined using GC–MS (GCMS-QP2020, Shimadzu, Kyoto, Japan). The soil samples were immediately stored at −80 °C until DNA extraction using the FastDNA SPIN kit for Soil (MP Biomedicals, Santa Ana, CA, United States) with two modifications, as described by Spohn et al. (2016). The resultant DNA was quantified by Picogreen assay (Invitrogen, Waltham, MA, USA) and the δ^18^O of the DNA was analyzed using a Delta V Plus IRMS (Thermo Scientific, Waltham, MA, USA) with a thermo-chemical elemental analyzer (TC/EA) and Conflo IV interface operating in continuous-flow mode. CUE was calculated as described by Geyer et al. (2019).


CUE=1−(kresp/kgrowth)

Where kresp and kgrowth represent the rate constants for respiration and growth, respectively, derived from 18O incorporation into CO₂ and microbial DNA.

### 2.6 DNA extraction and quantitative PCR

DNA was extracted from the soil (0.5 g) using the FastDNA SPIN kit for Soil (MP Biomedicals, Santa Ana, CA, United States) following the manufacturer’s instructions. The abundances of total bacteria and fungi were assessed by quantifying the bacterial and fungal ribosomal gene copy numbers using TB Green Premix (Takara Bio, Japan) and a Thermal Cycler Dice Real Time System Lite (Takara Bio, Japan). The bacterial 16S rRNA genes were estimated by primer pair 341F (5’-CCTACGGGNGGCWGCAG-3’)/785R(5’-GACTACHVGGGTATCTAATCC-3’) (Klindworth et al., 2013) under the following conditions: 4 min of initial denaturation at 95 °C, followed by 40 cycles with 40 s of denaturation at 94 °C, 30 s of annealing at 52 °C, 40 s of elongation at 72 °C, and a final extension at 72 °C for 7 min. Quantification of fungal 18S rRNA genes was conducted by primer set FR1 (5’-AICCATTCAATCGGTAIT-3’)/FF390 (5’-CGATAACGAACGAGACCT-3’) (Vainio and Hantula, 2000) with an initial step of 8 min of denaturation at 95 °C, a second step corresponding to 30 cycles of 30 s at 94 °C, 45 s at 50 °C for hybridization, and an elongation step of 2 min at 72 °C. The arbuscular mycorrhizal fungal (AMF) community was quantified based on the 18S rRNA gene fragments. Primers AMG1F (5’–ATAGGGATAGTTGGGGGCA T–3’) (Hewins et al., 2015) and AM1 (5’-GTTTCCCGTAAGGCGCCGAA-3’) (Helgason et al., 1998) were used with the following PCR conditions: 95 °C for 5 min, 35 cycles of denaturation at 95 °C for 15 s, annealing at 62 °C for 60 s, and extension at 72 °C for 20 s. For each sample, three independent qPCR assays were performed for each gene. qPCR values are reported as gene copy number g^−1^ dry soil.

### 2.7 High-throughput sequencing analysis

Bacterial 16 s rRNA V4–V5 genes and the ITS1 region of the fungal rRNA gene were amplified using the primer pairs 518F (5’-CCAGCAGCYGCGGTA-3’) and 926R (5’-CCGTCAATTCNTTTRAGT-3’), and 18SF (5’- GTAAAAGTCGTAACAAGGTTTC-3’) - 5.8SR (5’-GTTCAAAGAYTCGATGAT TCAC-3’), respectively, sequenced on the Illumina MiSeq platform (Macrogen) using 2 × 300 bp paired-end reads. All obtained sequences were analyzed following the methods described by Kang et al. (2023). The imported forward and reverse sequences were demultiplexed and filtered using plugins implemented in Quantitative Insights into Microbial Ecology (QIIME2 v2020.11) (Bolyen et al., 2019). Using these data, an amplicon sequence variant (ASV) table was built, in which chimeric sequences were identified and removed. Taxonomic assignment of ASVs was performed using the SILVA (version 138.1) and UNITE fungal ITS databases (version 8.3) for 16 s rRNA and fungal ITS, respectively (Nilsson et al., 2019; Quast et al., 2013). The sequences were deposited in the NCBI Sequence Read Archive under accession number PRJNA1344597.

### 2.8 Statistical analyses

All statistical analyses were performed using the R software (Development Core Team, 2013). Normal data distributions were evaluated using the Shapiro–Wilk normality test. A one-way analysis of variance (ANOVA) was conducted to evaluate the impact of the organic farming chronosequence on soil biotic and abiotic properties relative to conventional farming. The means were compared using Duncan’s test. Since most parameters did not meet normality assumptions even after transformation, generalized linear models (GLM) with appropriate error distributions were used to assess the effects of farming duration and crop type. Non-metric multidimensional scaling (NMDS) was performed to describe bacterial and fungal community structures based on the Bray–Curtis dissimilarity index. The significance of compositional differences among the groups was tested using an analysis of similarity (ANOSIM) with 999 permutations. Depending on data normality, either ANOVA or the Kruskal–Wallis test was performed to identify ASVs that showed significant changes in relative abundance with organic farming duration. The top 20 most abundant ASVs were selected as responsive ASVs.

The correlation coefficients between microbial CUE and bacterial amplicon sequence variants were calculated using Spearman’s rank correlation. The correlation between soil biological and biochemical parameters was assessed using the Spearman method and the corrplot package in R.

## 3 Results

### 3.1 Progressive soil carbon accumulation and stabilization under long-term organic farming

**Figure 1:**
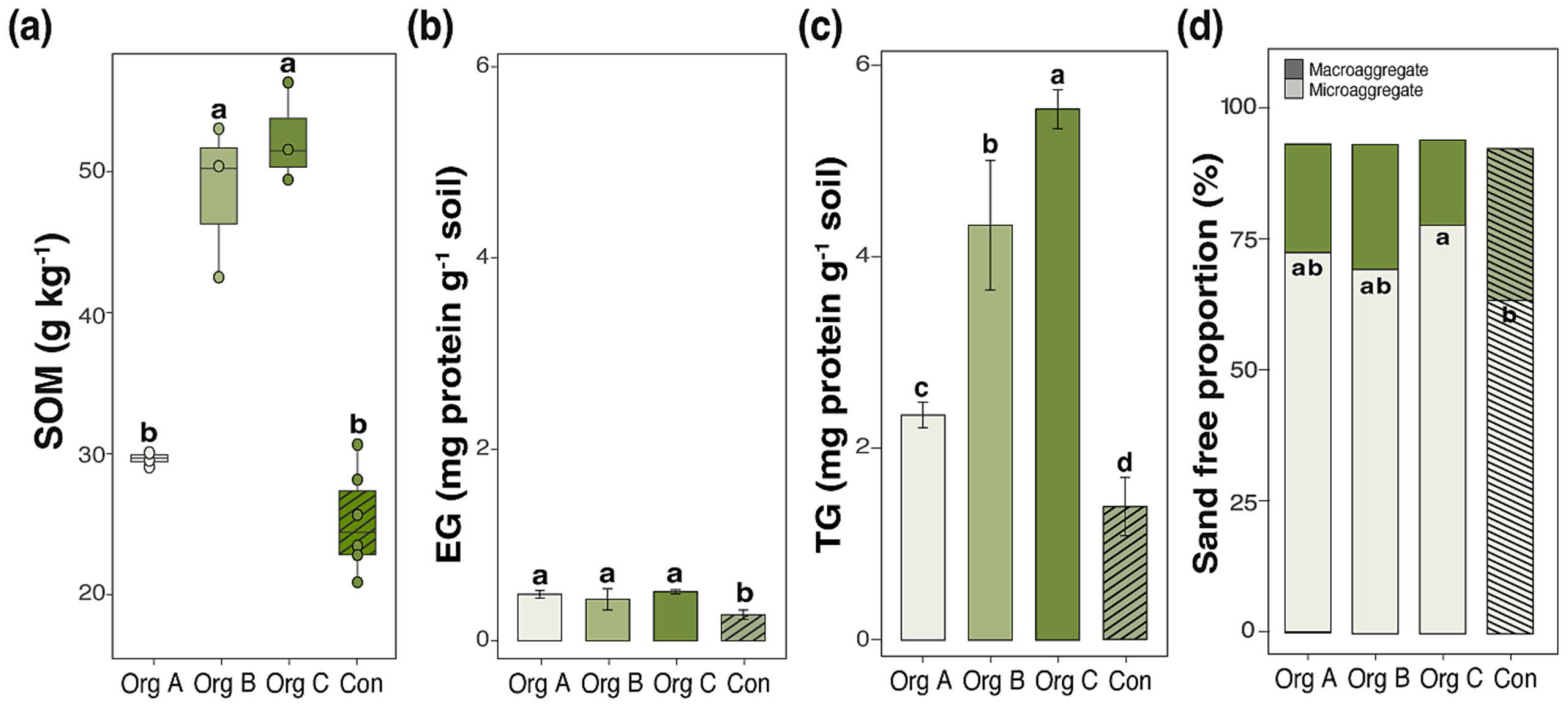
Effects of organic farming duration on soil organic matter, glomalin-related soil protein fractions, and aggregate distribution in cabbage cultivation systems. **(a)** Soil organic matter (SOM) content, **(b)** easily extractable glomalin (EG), **(c)** total glomalin (TG), and **(d)** sand-free water-stable aggregate distribution showing the proportion of macroaggregates (> 250 μm) and microaggregates (53–250 μm). Different lowercase letters indicate significant differences among treatments (Duncan test, *p* < 0.05). Error bars represent standard deviations.

Long-term organic management induced systematic changes in the soil carbon storage capacity, with distinct thresholds emerging across the farming duration gradient. The SOM content in cabbage systems demonstrated a progressive increase from 25.2 ± 3.7 g kg^−1^ under Con to 52.5 ± 3.5 g kg^−1^ in fields with Org C, representing a 108% enhancement (Table 1; Figure 1a). Generalized linear modeling (GLM) revealed that the organic farming duration was the dominant factor controlling SOM accumulation (*F* = 61.29, *p* < 0.001), explaining significantly more variation than crop type (*F* = 33.84, *p* < 0.001). This accumulation pattern exhibited non-linear dynamics with no significant changes during the initial 5 years (Org A: 29.6 ± 0.5 g kg^−1^), moderate increases at 5–10 years (Org B: 48.6 ± 5.6 g kg^−1^), and substantial accumulation beyond the 10-year threshold. Parallel patterns emerged in the pepper systems, although with lower absolute values, indicating a crop-mediated modulation of organic matter dynamics (Supplementary Figure S1a).

**Table 1:** Physicochemical properties of soils under different organic farming durations and conventional management in pepper and cabbage cultivation systems.

Crop	Cabbage				Pepper			
Label	Org A (*n* = 3)	Org B (*n* = 3)	Org C (*n* = 3)	Con (*n* = 6)	Org A (*n* = 3)	Org B (*n* = 3)	Org C (*n* = 3)	Con (*n* = 6)
Sand (%)	64.9 ± 1.6^ab^	60.5 ± 0.8^b^	52.6 ± 0.9^c^	68.4 ± 5.4^a^	75.3 ± 0.0^a^	67.5 ± 3.8^b^	49.8 ± 4.5^c^	76.6 ± 2.6^a^
Silt (%)	30.9 ± 1.6^b^	35.7 ± 1.9^a^	40 ± 0.9^a^	28.3 ± 4.9^c^	23.7 ± 0.7^c^	27.4 ± 2.2^b^	41.5 ± 3.0^a^	19.2 ± 1.9^d^
Clay (%)	4.2 ± 0.0^b^	3.8 ± 2.6^b^	7.4 ± 0.2^a^	3.3 ± 1.9^b^	1.0 ± 0.6^c^	5.1 ± 1.9^b^	8.8 ± 2.1^a^	4.2 ± 0.8^b^
pH	5.7 ± 0.3^a^	4.9 ± 0.1^b^	6.0 ± 0.1^a^	5.5 ± 0.6^ab^	4.4 ± 0.1^c^	5.2 ± 0.3^bc^	6.1 ± 0.1^a^	5.3 ± 0.6^ab^
Water content (%)	19.4 ± 0.7^b^	25.9 ± 2.0^a^	28.1 ± 0.6^a^	19.3 ± 2.2^b^	18.3 ± 1.1^c^	21.1 ± 0.8^b^	24.2 ± 1.7^a^	14.5 ± 1.0^d^
WHC (%)	70.1 ± 17.3^b^	70.3 ± 10.0^b^	90.9 ± 7.5^a^	55.2 ± 13.4^c^	52.7 ± 1.8^b^	58.6 ± 5.8^b^	69.9 ± 5.8^a^	43.6 ± 14.1^c^
NH_4_-N (mg kg^−1^)	41.7 ± 1.8	45.7 ± 1.8	42.4 ± 1.1	43.2 ± 4.6	83.4 ± 7.3^a^	45.2 ± 0.2^b^	44.2 ± 2.1^b^	40.6 ± 2.2^b^
NO_3_-N (mg kg^−1^)	55.9 ± 7.9^a^	45.9 ± 1.9^b^	42.8 ± 1.0^b^	45.0 ± 5.3^b^	115.4 ± 3.8^a^	105.2 ± 8.8^a^	54.6 ± 10.8^b^	50.2 ± 7.2^b^
TN (%)	0.1 ± 0.0^c^	0.2 ± 0.0^b^	0.3 ± 0.0^a^	0.1 ± 0.0^d^	0.1 ± 0.0^b^	0.2 ± 0.0^a^	0.2 ± 0.0^a^	0.1 ± 0.0^b^
TC (%)	1.2 ± 0.2^c^	2.6 ± 0.5^b^	3.8 ± 0.3^a^	0.9 ± 0.2^c^	0.8 ± 0.1^c^	1.5 ± 0.3^b^	3.2 ± 0.3^a^	0.7 ± 0.4^c^
CN	8.1 ± 1.6	11.2 ± 1.8	11.8 ± 1.5	9.5 ± 2.6	8.4 ± 1.8^b^	8.8 ± 1.4^b^	17.8 ± 1.9^a^	8.0 ± 3.1^b^
SOM (g kg^−1^)	29.6 ± 0.5^b^	48.6 ± 5.6^a^	52.5 ± 3.5^a^	25.2 ± 3.7^b^	23.7 ± 1.5^c^	31.8 ± 1.1^b^	39.6 ± 3.8^a^	21.5 ± 3.2^c^
Available P (mg kg^−1^)	1,224.1 ± 291.3^b^	2,804.6 ± 129.8^a^	2,661.0 ± 37.5^a^	619.2 ± 161.3^c^	340.0 ± 101.7^b^	1,240.8 ± 153.8^a^	861.8 ± 224.2^a^	334.5 ± 235.1^b^
Ca (cmol_c_ kg^−1^)	5.7 ± 1.0^b^	5.3 ± 0.4^b^	10.5 ± 0.1^a^	7.1 ± 1.4^b^	6.2 ± 0.2^b^	6.4 ± 1.1^b^	10.0 ± 0.5^a^	8.4 ± 1.1^a^
K (cmol_c_ kg^−1^)	0.5 ± 0.1^b^	0.7 ± 0.0^a^	0.8 ± 0.1^a^	0.3 ± 0.1^c^	0.3 ± 0.0^b^	0.7 ± 0.0^a^	0.3 ± 0.0^b^	0.3 ± 0.1^b^
Mg (cmol_c_ kg^−1^)	1.8 ± 0.4^b^	1.0 ± 0.2^c^	3.2 ± 0.1^a^	1.9 ± 0.6^b^	1.6 ± 0.1^c^	2.3 ± 0.1^bc^	3.6 ± 0.3^a^	3.2 ± 0.7^ab^
Na (cmol_c_ kg^−1^)	0.4 ± 0.1^a^	0.5 ± 0.0^a^	0.5 ± 0.0^a^	0.3 ± 0.0^b^	0.7 ± 0.1^b^	1.2 ± 0.2^a^	0.5 ± 0.0^b^	0.4 ± 0.1^b^
CEC (cmol_c_ kg^−1^)	10.4 ± 0.9^c^	12.3 ± 0.1^b^	15.9 ± 0.1^a^	11.7 ± 0.9^b^	10.6 ± 0.4^b^	12.4 ± 0.9^ab^	15.1 ± 1.0^a^	13.8 ± 1.8^ab^

Values are means ± standard deviations. Org A, Org B, and Org C represent organic farming practices with durations of 0–5, 5–10, and > 10 years, respectively. Con represents conventional farming practice. Different lowercase letters within each crop type indicate significant differences among treatments based on one-way ANOVA with the Duncan test as a post-hoc test (*p* < 0.05). WHC, water holding capacity; TN, total nitrogen; TC, total carbon; CN, carbon to nitrogen ratio; SOM, soil organic matter; CEC, cation exchange capacity.

The differential accumulation patterns of the GRSP fractions revealed mechanistic insights into carbon stabilization processes. While EG remained relatively stable across treatments in cabbage systems (0.43–0.51 mg protein g^−1^ soil; Figure 1b), the TG content increased 4.0-fold from Con (1.4 ± 0.3 mg protein g^−1^ soil) to Org C (5.6 ± 0.2 mg protein g^−1^ soil; Figure 1c), showing definite variation (*F* = 34.24, *p* < 0.001).

Soil aggregate distribution patterns provide physical evidence for enhanced carbon protection mechanisms. Microaggregate proportions remained stable (71.7–74.8%) across Org A and B, but showed a significant decline only in Con (65.6 ± 6.4%), coinciding with increased macroaggregate formation (Figure 1d). The GLM confirmed the significant effect of organic management on soil aggregation with no significant crop and management interactions, suggesting consistent aggregate stabilization across crop types.

TC content exhibited enrichment patterns increasing from 0.9 ± 0.2% in Con to 3.8 ± 0.3% in Org C. The GLM revealed highly significant effects of organic matter duration on TC accumulation (*F* = 30.23, *p* < 0.001), with crop type showing a secondary influence (*F* = 6.55, *p* < 0.05; Supplementary Table S1). The carbon to nitrogen ratios (TC: TN) remained relatively stable across treatments despite substantial carbon accumulation indicating coupled nutrient cycling rather than simple organic matter accumulation. This stoichiometric preservation was further supported by parallel increases in TN content (0.1 to 0.3%) and significant organic duration effects on both the NH_4_-N (*F* = 33.32, p < 0.001) and NO_3_-N (*F* = 39.00, *p* < 0.001) pools. Available phosphorus increased significantly with organic farming duration, rising from 619.2 ± 161.3 mg kg^−1^ in Con to 2,661.0 ± 37.5 mg kg^−1^ in Org C for cabbage systems (Table 1). This 4.3-fold increase in plant-available phosphorus, coupled with the observed shifts in enzymatic stoichiometry.

WHC increased concomitantly with organic matter content reaching 90.9 ± 7.5% in Org C compared to 55.2 ± 13.4% in Con. The strong organic duration effect on WHC (*F* = 37.68, *p* < 0.001), coupled with significant crop effects (*F* = 44.91, *p* < 0.001), demonstrated the differential soil physical property responses between the cultivation systems. Cation exchange capacity similarly responded to organic management duration (*F* = 19.97, *p* < 0.001), increasing from 11.7 ± 0.9 to 15.9 ± 0.1 cmolc kg^−1^ in cabbage systems.

### 3.2 Organic farming duration drives distinct microbial succession patterns

**Figure 2:**
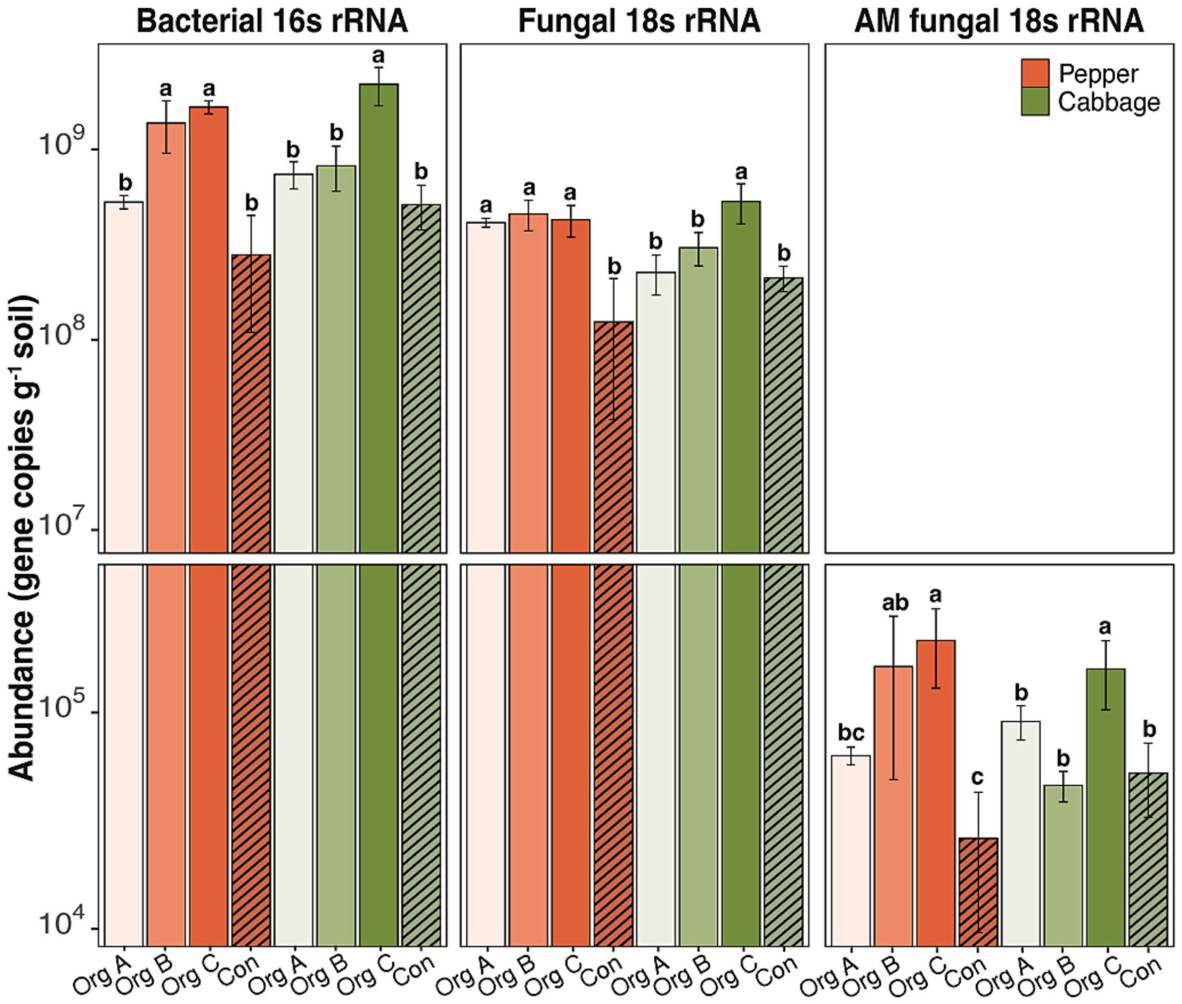
Abundance of bacterial, fungal, and arbuscular mycorrhizal fungal communities in soils under different organic farming durations in pepper and cabbage cultivation systems. Values are means ± standard deviations. Different lowercase letters indicate significant differences among treatments within each crop type (Duncan test, *p* < 0.05). Note the logarithmic scale for bacterial and total fungal abundance.

The observed carbon accumulation patterns corresponded to the systematic restructuring of soil microbial communities along the organic farming chronosequence. Quantitative PCR analyses demonstrated distinct and contrasting temporal dynamics in the bacterial and fungal populations across the management systems. Bacterial 16S rRNA gene abundance in cabbage soils markedly increased under organic management, reaching its highest level in Org C (2.2 × 10^9^ gene copies g^−1^ soil) while declining significantly in Con (5.1 × 10^8^ gene copies g^−1^ soil; Figure 2). Similarly, the fungal 18S rRNA gene abundance also increased significantly across all organic management (2.2–5.3 × 10^8^ gene copies g^−1^ soil) compared to Con (2.1 × 10^8^ gene copies g^−1^ soil; *p* < 0.05). The shift toward fungal dominance was particularly pronounced for AMF which increased 3-fold from Con (5.2 × 10^4^ gene copies g^−1^) to Org C (1.6 × 10^5^ gene copies g^−1^), paralleling the observed GRSP accumulation patterns and suggesting potential associations between AMF abundance and carbon stabilization mechanisms.

**Figure 3:**
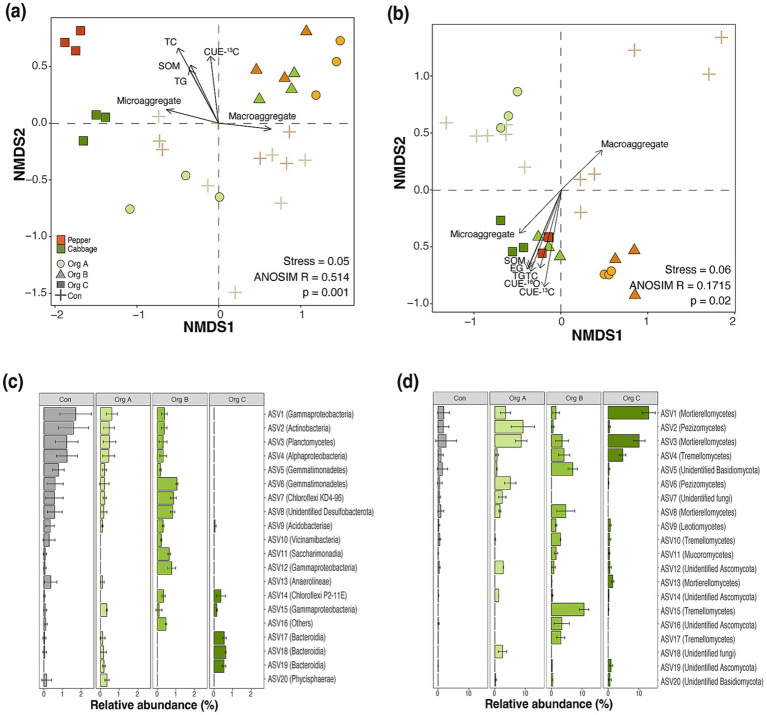
Microbial community structure and composition shifts along organic farming practice duration in cultivation systems. Non-metric multidimensional scaling (NMDS) ordination based on Bray–Curtis dissimilarity showing **(a)** bacterial community structure and **(b)** fungal community structure. Vectors represent significant environmental variables correlated with community composition (*p* < 0.05). Stress values and analysis of similarity (ANOSIM) statistics indicate ordination quality and significance of community differences among treatments. Relative abundance of the top 20 most abundant **(c)** bacterial and **(d)** fungal amplicon sequence variants (ASVs) that showed significant responses to organic farming duration (one-way ANOVA or the Kruskal–Wallis test, *p* < 0.05) in the cabbage cultivation system. ASVs are classified at the phylum level in parentheses.

The NMDS ordination revealed distinct community assembly patterns driven by the duration of organic farming. Bacterial communities exhibited clear temporal clustering, with Con and Org C occupying opposite extremes along NMDS1 (ANOSIM R = 0.514, *p* = 0.001; Figure 3a). Environmental fitting identified TC, SOM, TG, and CUE-^13^C as significant drivers of bacterial community structure, suggesting tight coupling between community composition and carbon cycling functions (*p* < 0.05). Fungal communities demonstrated weaker differentiation, with carbon cycling functions emerging as the primary structuring factor, indicating the fungal mediation of soil physical properties (ANOSIM *R* = 0.1715, *p* = 0.02; Figure 3b).

Differential abundance analysis of the ASVs revealed taxonomically coherent succession patterns underlying community restructuring. Among the top 20 responsive bacterial ASVs in cabbage systems, Con were dominated by Gammaproteobacteria, Actinobacteria, Plantomycetes, and Alphaproteobaceria (combined relative abundance: 9.8 ± 3.8%; Figure 3c; Supplementary Table S2). This dominance progressively declined with organic farming duration, reaching only 2.3 ± 0.4% in Org C. Conversely, Bacteroidia (ASV17, ASV18, and ASV19) and Chloroflexi (ASV14) increased from a negligible presence in Con. Parallel bacterial succession patterns emerged in the pepper cultivation systems with distinct taxonomic compositions (Supplementary Figure S2a). Notably, Nitrospira (ASV16 and ASV17) emerged as unique responders in pepper systems, but were absent in cabbage, indicating a crop-specific enrichment of nitrogen-cycling taxa. Suggesting crop-specific selection for nitrogen cycling taxa.

Fungal community succession exhibited distinct phylogenetic patterns, with Mortierellomycetes emerging as the dominant taxon. Three Mortierellomycota ASVs (ASV1, ASV3, and ASV13), Tremellomycetes (ASV4), and unidentified Ascomycota (ASV19) collectively increased from 5.4 ± 0.0% in Con to 30.6 ± 4.1% in cabbage Org C (Figure 3d). Additional fungal responders included members of Mortierellomycota (ASV 8), Tremellomycetes (ASV15 and ASV17), unidentified Ascomycota (ASV16), and unidentified Basidiomycota (ASV5), which demonstrated intermediate response patterns with maximum abundance in Org B. Pepper cultivation systems exhibited parallel, but amplified, patterns with Mortierellomycetes reaching 60.8 ± 3.9% relative abundance in Org C (Supplementary Fig S2b).

The cumulative relative abundance patterns revealed contrasting dynamics between the bacterial and fungal communities. Bacterial-responsive ASVs demonstrated modest compositional shifts, with cumulative abundance ranging from 1.2 ± 1.0% in Org C to 9.8 ± 3.8% in Con. In contrast, fungal communities exhibited dramatic restructuring. Mortierellomycetes emerged as the dominant responsive taxon across both crop types with three Mortierellomycetes collectively increasing from 4.4 ± 1.9% to 24.9 ± 2.1% in cabbage (Figure 3d) and reaching 50.8 ± 2.3% relative abundance in pepper Org C (Supplementary Figure S2b). The magnitude of fungal restructuring, characterized by a 5.6- to 7.4-fold increase in Mortierellomycete abundance, contrasted sharply with the relatively subtle bacterial compositional changes, suggesting that organic farming exerts stronger selective pressure on fungal communities.

### 3.3 Microbial metabolic efficiency enhancement as a key mechanism for carbon stabilization

**Figure 4:**
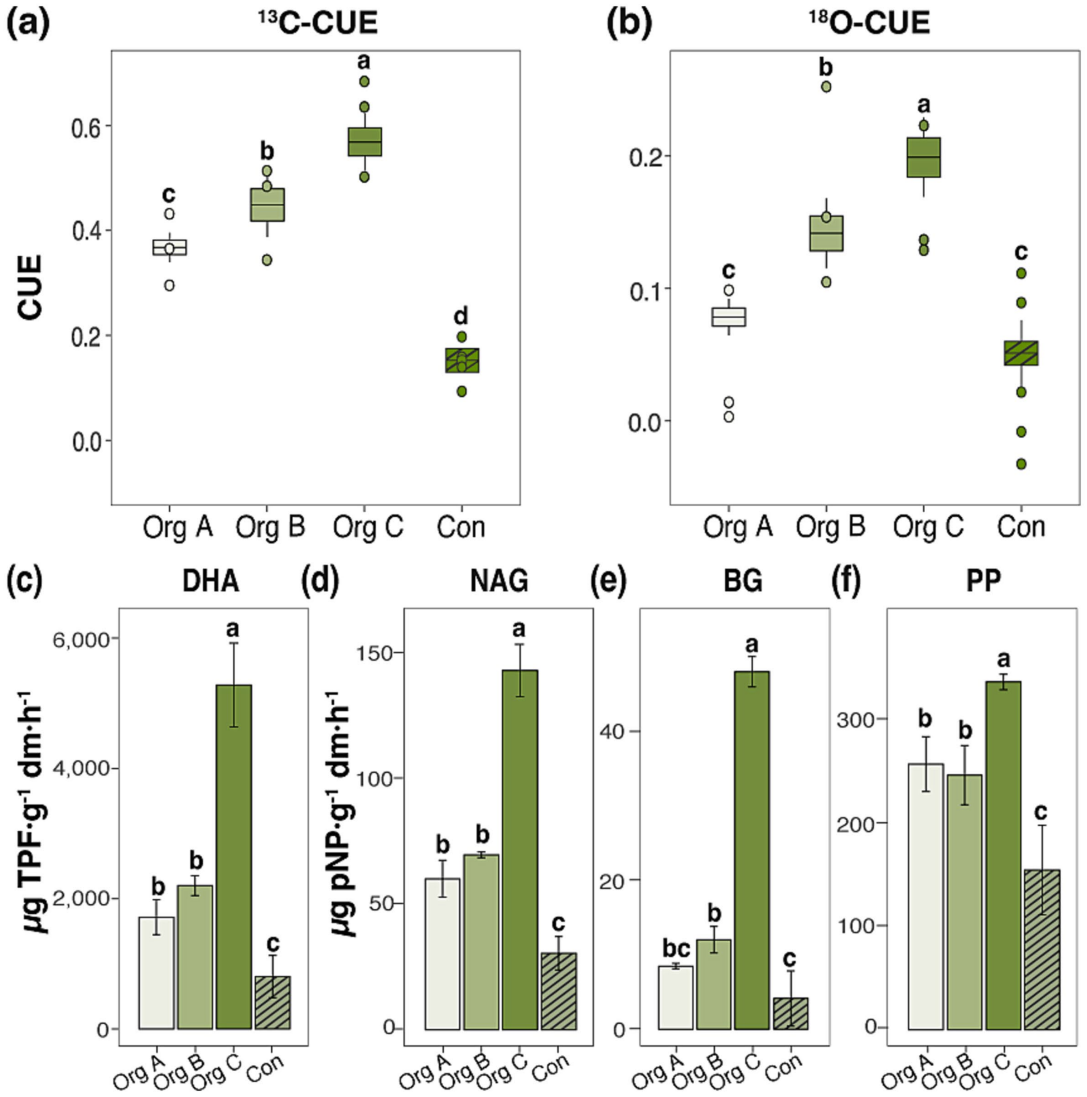
Microbial carbon use efficiency and extracellular enzyme activities in cabbage cultivation systems under different organic farming durations. Microbial carbon use efficiency (CUE) measured using **(a)** ^13^C-glucose and **(b)** ^18^O-H_2_O labeling methods. Soil extracellular enzyme activities: **(c)** Dehydrogenase activity (DHA), **(d)** *N*-acetyl-β-glucosaminidase (NAG), **(e)** β-glucosidase (BG), and **(f)** acid phosphatase (PP). Different lowercase letters indicate significant differences among treatments (Duncan test, *p* < 0.05).

The observed restructuring of the microbial community corresponds to alterations in the metabolic efficiency and enzymatic activities along the organic farming chronosequence. CUE measurements using dual isotopic approaches revealed a progressive enhancement of the microbial metabolic efficiency with increasing duration of organic management. In cabbage systems, ^13^C-CUE increased from 0.15 ± 0.02 in conventional soils to 0.56 ± 0.05 in Org C (Figure 4a), representing a 3.7-fold enhancement. The ^18^O-H₂O method corroborated these patterns with CUE values increasing from 0.05 ± 0.01 to 0.20 ± 0.02 along the same gradient (Figure 4b). Pepper systems exhibited higher ^13^C-CUE and ^18^O-H₂O under organic management than under Con. However, the values did not display a linear increase with the organic farming duration compared to near-zero levels under conventional management (Supplementary Figures S3a,b).

Extracellular enzyme activity exhibits crop-specific responses to organic farming duration. DHA, an indicator of total microbial metabolic activity, increased 6.6-fold from conventional (803.5 ± 324.0 μg TPF g^−1^ dm h^−1^) to Org C (5,279.5 ± 643.3 μg TPF g^−1^ dm h^−1^) in cabbage (Figure 4c). BG demonstrated the most pronounced enhancement increasing from 4.1 ± 3.7 to 47.8 ± 2.0 μg pNP g^−1^ dm h^−1^ (Figure 4e). NAG and PP showed parallel increases, reaching their maximum activity in Org C (Figures 4d,f). Pepper plants exhibited similar enzymatic enhancement patterns, although distinct intermediate responses were observed for Org B (Supplementary Figures S3c–f).

Correlation analyses between the microbial CUE and specific ASV abundance revealed taxonomically coherent metabolic strategies underlying efficiency enhancement. Among the bacterial taxa in cabbage systems, 13 out of 20 responsive ASVs demonstrated significant negative correlations with ^13^C-CUE (Supplementary Table S4). Conversely, specific Chloroflexi members (ASV14) and Bacteroidetes representatives (ASV17-19) were positively associated with enhanced CUE. Pepper bacterial communities revealed distinct metabolic associations with Acidobacteria (ASV3-4, 10, 12), exhibiting consistently positive CUE correlations (rho = 0.57–0.63; Supplementary Table S4). The emergence of Nitrospira as a negatively correlated taxa (rho = −0.13 to −0.07) suggested crop-specific selection for nitrogen-cycling organisms with reduced CUE.

Fungal taxa demonstrated contrasting metabolic associations with 16 out of 20 responsive ASVs, showing positive correlations with ^13^C-CUE in cabbage systems (Supplementary Table S5). Mortierellomycetes exhibited consistently strong positive relationships (rho = 0.670.71), and specific Tremellomycetes (rho = 0.58–0.66). These positive associations persisted in pepper systems where Mortierellomycetes ASVs maintained robust correlations with CUE (rho = 0.56–0.77; Supplementary Table S6) while Mucoromycota demonstrated the strongest relationship (rho = 0.59–0.77).

**Figure 5:**
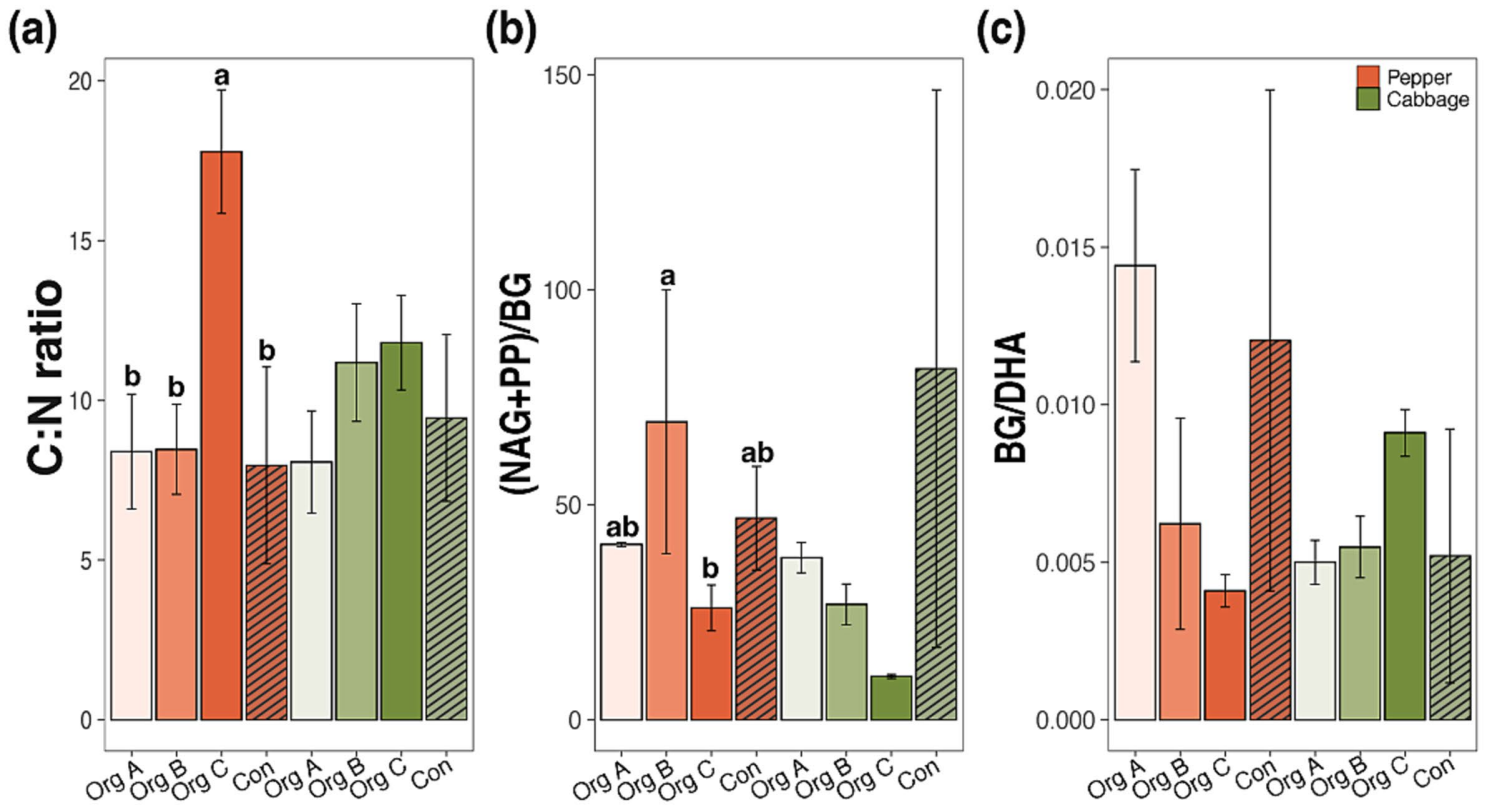
Soil stoichiometry and enzymatic balance indicators in pepper and cabbage cultivation systems under different organic farming durations. **(a)** Soil carbon to nitrogen ratio (C:N), **(b)** Eco-enzymatic stoichiometry expressed as the ratio of nitrogen (NAG) and phosphorus (PP) acquisition enzymes to the carbon acquisition enzyme (BG), calculated as (NAG+PP)/BG, and **(c)** The ratio of β-glucosidase to dehydrogenase activity (BG/DHA) representing the carbon acquisition efficiency relative to the total microbial metabolic activity. Bars represent means ± standard deviations. Different lowercase letters indicate significant differences among treatments within each crop type (Duncan test, *p* < 0.05). NAG, *N*-acetyl-β-glucosaminidase; PP, acid phosphatase; BG, β-glucosidase; DHA, dehydrogenase activity.

Enzymatic stoichiometry analyses provide mechanistic insights into nutrient cycling alterations that accompany metabolic efficiency enhancement. The CN increased significantly only in pepper Org B systems (17.8 ± 1.9) while maintaining relative stability in cabbage fields (8.1–11.8; Figure 5a). Eco-enzymatic stoichiometry, calculated as (NAG + PP)/BG, decreased progressively with the organic farming duration in both crops from 81.7 ± 64.8 in Con to 10.1 ± 0.5 in Org C cabbage systems (Figure 5b). This shift indicates a reduced investment in nitrogen and phosphorus acquisition relative to carbon cycling enzymes, suggesting the alleviation of nutrient limitations under organic management. The BG/DHA ratio declined compared to that of Con, with a particularly pronounced decrease in the cabbage field of Org C, where extended organic management led to a progressive reduction over time (Figure 5c). The 3.8-fold enhancement in CUE, coupled with increased enzymatic activity and shifts toward carbon-acquisition enzymes, demonstrated that long-term organic management fundamentally alters soil metabolic networks toward enhanced carbon retention capacity.

### 3.4 Integrated biological-biochemical pathways mediating soil carbon sequestration

**Figure 6:**
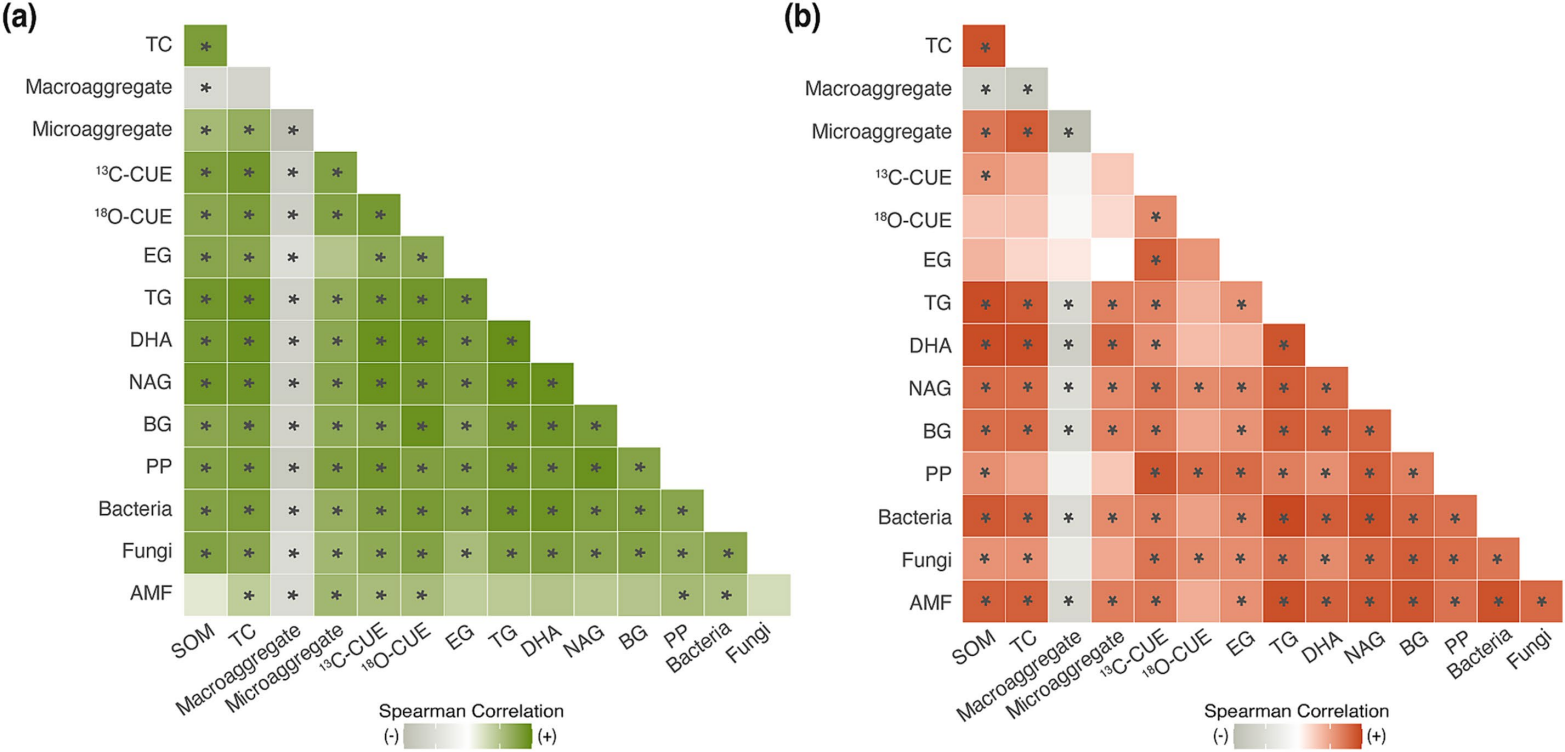
Spearman correlation matrices of soil biological and biochemical parameters in **(a)** cabbage and **(b)** pepper systems. Correlations among carbon fractions (SOM and TC), aggregates, microbial efficiency (^13^C-CUE, and ^18^O-CUE), glomalin (EG and TG), enzyme activities (DHA, NAG, BG, and PP), and microbial abundance (bacteria, fungi, and AMF). Asterisks indicate significant correlations (*p* < 0.05). Color intensity represents correlation strength.

Correlation analyses revealed a complete mechanistic pathway linking the duration of organic farming to enhanced carbon stabilization. The Spearman correlation matrices demonstrated highly integrated networks, with most pairwise correlations achieving significance in both crop systems (Figures 6a,b). CUE measurements showed particularly strong correlations with carbon fractions with ^13^C-CUE exhibiting significant positive relationships with SOM (rho = 0.83), TC (rho = 0.89), and TG (rho = 0.88) in the cabbage system. The ^18^O-CUE showed comparable correlations (*ρ* = 0.75–0.90), confirming the consistency of metabolic efficiency enhancement across measurement approaches.

GRSP fractions have emerged as central integrators of soil biological and physical properties (Singh et al., 2024). TG demonstrated robust correlations with bacterial and fungal abundance (rho = 0.79–0.91) and all measured enzyme activities (rho = 0.89–0.96) in cabbage systems. TG showed strong positive correlations with microaggregates, but consistent negative correlations with macroaggregates across both crops. In contrast, EG was weakly positive for macroaggregates in pepper, but negative for cabbage, indicating no significant relationship with microaggregates. Microaggregate formation exhibited significant positive correlations with both ^13^C-CUE in cabbage soils (rho = 0.80), indicating the metabolic efficiency-aggregation relationship, whereas no significant relationship was observed in pepper soils (rho = 0.29).

Microbial abundance demonstrated differential relationships with soil properties between crop systems. In cabbage fields, bacterial and fungal abundances showed comparable correlations with carbon fractions (bacteria-SOM: rho = 0.79, fungi-SOM: rho = 0.8; Figure 6a), whereas pepper systems exhibited stronger bacterial associations (bacteria-SOM: rho = 0.89, fungi-SOM: rho = 0.58; Figure 6b). The AMF abundance showed a significant positive correlation with TG in pepper systems (*p* < 0.05), but not in cabbage systems, suggesting crop-specific mechanisms for GRSP production.

## 4 Discussion

Our study identified a critical 10-year threshold for achieving substantial soil carbon sequestration under organic management with a lag phase showing no significant accumulation during the initial 0–5 years, followed by accelerated storage beyond this temporal boundary. While this pattern aligns with recent meta-analyses showing extended timeframes for carbon accumulation under organic management (Beillouin et al., 2023; Mgelwa et al., 2025), validation across diverse cropping systems, climatic zones, and soil types is needed to determine the generalizability of this threshold. The 108% increase in SOM and 4.0-fold enhancement in TG after 10 years of organic practices aligns with recent meta-analyses showing that significant carbon accumulation requires extended management periods (Beillouin et al., 2023; Mgelwa et al., 2025). However, our study provides mechanistic explanations for these temporal dynamics through microbial succession patterns. The observed lag phase with no significant accumulation (0–5 years), followed by rapid accumulation (5–10 years) and stabilization (> 10 years), reflects fundamental shifts in soil biological processes rather than simple organic matter addition. This pattern corresponds with recent findings by Tian et al. (2024), who demonstrated that restructuring of microbial community precedes measurable carbon accumulation over several years. However, our identification of specific microbial indicators (Mortierellomycete proliferation and Gammaproteobacteria decline) provides actionable biomarkers for monitoring the transition progress. The divergence between the EG and TG pools over time revealed progressive carbon stabilization mechanisms in the soil. While Zhang et al. (2023) reported similar GRSP accumulation patterns, our demonstration of increasing TG/EG ratios from 4.1 to 10.6 quantifies the shift toward recalcitrant carbon forms. This finding suggests that the duration of organic farming influences not only the quantity, but also the quality of stored carbon.

Our finding that CUE increased 3.7-fold under long-term organic management in cabbage systems represents a paradigm shift in our understanding of soil carbon sequestration mechanisms. Although previous studies have measured CUE changes under different management conditions (He et al., 2024), this study is the first to directly link specific microbial taxa to CUE variations during organic transitions. Dual isotope validation, showing consistent patterns, strengthens the confidence in these metabolic shifts, addressing the methodological concerns raised by Yang et al. (2025) regarding single-method CUE measurements. Taxonomic coherence in CUE relationships with Gammaproteobacteria consistently showed negative correlations, whereas those with Mortierellomycetes demonstrated positive associations, providing mechanistic insights into community-level metabolic changes. This pattern aligns with recent theoretical frameworks that distinguish copiotrophic and oligotrophic life strategies (Ma et al., 2023), but extends these concepts by quantifying strategy-specific contributions to ecosystem-level carbon cycling. The dominance of Mortierellomycetes in high-CUE systems supports the emerging evidence that fungi, particularly Mortierellomyceta, possess superior carbon conversion efficiency compared to bacteria (Wu et al., 2025). The superior carbon conversion efficiency of Mortierellomycetes can be attributed to several metabolic characteristics. First, Mortierellomycetes possess extensive lipid biosynthesis pathways, allowing efficient conversion of carbohydrates into storage lipids with minimal respiratory losses compared to bacterial carbohydrate metabolism (Wu et al., 2025). Second, as oligotrophic fungi, Mortierellomycetes exhibit slow growth rates with low maintenance respiration costs, allocating a greater proportion of assimilated carbon to biomass rather than energy generation (Ma et al., 2023). Third, their filamentous hyphal network enables efficient substrate exploration with lower energetic costs per unit biomass compared to bacterial motility and chemotaxis (Liu et al., 2024). These combined metabolic traits explain why Mortierellomycetes dominance correlates strongly with enhanced ecosystem-level CUE and carbon accumulation. Notably, our observation that bacterial abundance remained relatively stable while CUE increased dramatically demonstrates that metabolic efficiency plays a critical role in carbon sequestration beyond biomass quantity alone. Our results support the emerging view that both microbial biomass and carbon use efficiency jointly determine soil carbon formation (Tao et al., 2023), with metabolic efficiency representing a previously underappreciated mechanism. This finding suggests that models incorporating both microbial abundance and metabolic efficiency will better predict soil organic carbon dynamics than biomass-centric approaches alone. The shift from inefficient Gammaproteobacteria to efficient fungal taxa represents a fundamental reorganization of soil metabolic networks optimized for carbon retention. Beyond taxonomic shifts in CUE, our hypothesis predicted that metabolic efficiency enhancement would be accompanied by coordinated changes in enzymatic investment strategies.

The coordinated enhancement of enzyme activity and CUE improvements revealed integrated metabolic optimization under organic management. Our finding that the eco-enzymatic stoichiometry [(NAG + PP)/BG] decreased from 47.5 to 10.1 indicates a fundamental shift in microbial nutrient acquisition strategies, which is consistent with reduced nutrient limitation in organic systems. This pattern extends the recent study by Abay et al. (2024) on enzymatic stoichiometry, offering the novel insight that CUE enhancement precedes and potentially drives enzymatic rebalancing. The strong correlations between CUE and all measured enzymes suggest that metabolically efficient communities invest more resources in extracellular enzymes, contradicting the assumption that a high CUE reduces enzymatic investment (He et al., 2024). Our data support an alternative model in which efficient metabolism generates surplus resources, enabling enhanced enzyme production while creating positive feedback loops for organic matter processing. The differential enzyme responses observed between crops, with cabbage showing uniform enhancement and pepper exhibiting more variable patterns, indicate plant-mediated modulation of microbial investment strategies. Recent rhizosphere studies (Ghaderi et al., 2025; Qu et al., 2022) have shown crop-specific effects on enzyme expression; however, our demonstration of CUE-enzyme coupling provides a unifying framework for understanding these variations. The smaller increase in BG/DHA ratios, despite an increase in absolute activity, suggests proportional scaling of carbon acquisition and total metabolism, supporting theoretical predictions of the optimal foraging theory applied to microbial communities (Manzoni et al., 2023). Finally, our hypothesis proposed that these metabolic pathways would be modulated by crop identity creating distinct carbon sequestration mechanisms.

The contrasting network architectures of cabbage and pepper cultivation systems reveal crop-specific carbon sequestration pathways with important management implications. Beyond root architecture differences, these crops likely differ in exudate chemistry. Pepper, as a mycorrhizal crop, preferentially stimulates AMF through lipid- and organic acid-rich exudates, resulting in strong AMF-GRSP correlations. Cabbage produces glucosinolate-rich exudates favoring saprotrophic fungi, particularly Mortierellomycetes, explaining non-mycorrhizal GRSP production pathways. This suggests that crop rotations alternating mycorrhizal and non-mycorrhizal species may maintain complementary fungal communities and enhance carbon sequestration through diverse stabilization mechanisms. This finding extends recent work on plant–soil feedback by demonstrating how crop identity influences the coupling strength between microbial community structure and function (Camli-Saunders and Villouta, 2025; Sharma et al., 2023). The absence of a direct AMF-GRSP correlation in cabbage, despite parallel increases, suggests the existence of alternative GRSP sources or indirect production pathways. Recent studies have identified non-mycorrhizal fungi and bacteria as potential GRSP producers (Irving et al., 2021; Son et al., 2024). Our strong Mortierellomycetes-glomalin associations in cabbage support this hypothesis. This crop-specific variation in GRSP sources has implications for inoculation strategies, suggesting that AMF amendments may be more effective in pepper systems, whereas cabbage may benefit from diverse fungal consortia. Our results indicate that crop selection and rotation strategies should consider carbon sequestration efficiency, along with traditional agronomic factors. The superior integration of cabbage systems suggests their potential as carbon-sequestering crops in rotation sequences, whereas the modular structure of pepper may offer resilience benefits under stress conditions. These findings align with carbon-smart agriculture, but provide specific and mechanically grounded recommendations for implementation (Yeboah et al., 2021).

Our integrated pathway, from organic management through microbial succession and metabolic enhancement to carbon stabilization, provides a mechanistic framework for predicting and optimizing soil carbon sequestration. The central role of CUE as a master regulator connecting community structure to ecosystem function represents a significant advancement over current models that treat microbial communities as black boxes (Connors et al., 2024). The 3.7-fold CUE enhancement, translating to a 4.0-fold carbon accumulation, suggests an efficiency transfer coefficient of approximately 1.1, providing a quantitative basis for incorporating microbial efficiency into Earth system models. The identification of Mortierellomycetes as a keystone taxon for carbon sequestration, which consistently correlated with enhanced CUE across both crop systems, offers practical targets for microbiome engineering. Recent advances in fungal inoculation technologies (Conti et al., 2025; Díaz-Urbano et al., 2023) have accelerated the organic transition. However, our observation of 10-year thresholds suggests that establishing stable and efficient communities requires extended selection pressures, which may not be replaceable by simple inoculation. Although our study provides robust mechanistic insights, several limitations warrant consideration. The focus on two crops in a single climatic zone limits the generalizability of our findings, particularly regarding the observed 10-year threshold for carbon accumulation. Future research should examine whether similar temporal thresholds and mechanistic pathways occur across diverse cropping systems, soil types, and climatic conditions to establish the universality of these patterns. Additionally, while our correlative approach identified strong associations, experimental manipulation of CUE through selective inhibition or isotope tracing would provide causal validation. Future research should employ metagenomics and metatranscriptomics to link taxonomic shifts with functional gene expression, particularly focusing on carbon metabolic pathways. Our finding that meaningful sequestration requires more than 10 years of continuous organic management highlights the need for long-term policy support and economic incentives to sustain these practices. The development of CUE-based indicators could provide rapid assessment tools for carbon credit verification, addressing the current challenges in monitoring soil carbon changes.

## Data Availability

The datasets presented in this study can be found in online repositories. The names of the repository/repositories and accession number(s) can be found in the article/Supplementary material.
