# Supplementary material for: Microbial efficiency enhancement drives carbon sequestration in long-term organic farming systems: linking taxonomic succession to carbon use efficiency
Source: Front Microbiol. 2026 Mar 16;17:1770908. doi: 10.3389/fmicb.2026.1770908 (PMC13033635; doi:10.3389/fmicb.2026.1770908)

**Microbial metabolic efficiency enhancement drives soil carbon sequestration after long organic farming: Linking taxonomic succession to carbon use efficiency**

Bo Ram Kang [a], Young Jun Bae [a], Soundarya Rajapitamahuni [a], Min-Seob Kim [b], Soon-Jae Lee [c], Youngmi Lee [d], Hong Shik Nam [d], Tae Kwon Lee [a,*]

[a] Department of Environmental and Energy Engineering, Yonsei University, Wonju 26493, Republic of Korea

[b] Fundamental Environment Research Department, Environmental Measurement & Analysis Center, National Institute of Environmental Research, Incheon, 22766, Korea

[c] Department of Ecology and Evolution, University of Lausanne, Lausanne 1015, Switzerland

[d] Organic Agriculture Division, National Institute of Agricultural Sciences, Wanju 55365, Republic of Korea

*** Corresponding author:**

E-mail addresses: tklee@yonsei.ac.kr (T. K. Lee)

Phone: +82-33-760-2446, Fax: +822-33-760-5286

**Table S1** Statistical analysis of soil physicochemical properties as influenced by organic farming duration and crop type using generalized linear models. Two-way ANOVA was performed to assess the individual and interactive effects of organic farming duration (0-5, 5-10, >10 years, and conventional) and crop type (pepper and cabbage) on soil parameters. The table presents F-statistics and associated P-values for main effects (Organic, Crop) and

interaction terms (Organic:Crop). WHC, water holding capacity; TN, total nitrogen; TC, total carbon; CN, C:N ratio; SOM, soil organic matter; CEC, cation exchange capacity.

| **Indicator** | **Term** | **F** | **P value** | **Indicator** | **Term** | **F** | **P value** |
|---|---|---|---|---|---|---|---|
| | Organic | 57.23 | p<0.001 | | Organic | 61.29 | p<0.001 |
| Sand | Crop | 16.29 | p<0.001 | SOM | Crop | 33.84 | p<0.001 |
| | Organic:Crop | 3.03 | 0.05 | | Organic:Crop | 1.84 | 0.17 |
| | Organic | 42.02 | p<0.001 | | Organic | 24.24 | p<0.001 |
| Silt | Crop | 42.01 | p<0.001 | Available P | Crop | 33.83 | p<0.001 |
| | Organic:Crop | 4.87 | p<0.05 | | Organic:Crop | 1.08 | 0.38 |
| | Organic | 10.65 | p<0.001 | | Organic | 20.53 | p<0.001 |
| Clay | Crop | 0.18 | 0.67 | Ca | Crop | 4.18 | 0.05 |
| | Organic:Crop | 4.83 | p<0.05 | | Organic:Crop | 0.98 | 0.42 |
| | Organic | 5.82 | p<0.01 | | Organic | 28.72 | p<0.001 |
| pH | Crop | 2.06 | 0.17 | K | Crop | 17.50 | p<0.001 |
| | Organic:Crop | 3.93 | p<0.05 | | Organic:Crop | 10.30 | p<0.001 |
| | Organic | 24.93 | p<0.001 | | Organic | 16.68 | p<0.001 |
| Water content | Crop | 22.83 | p<0.001 | Mg | Crop | 26.94 | p<0.001 |
| | Organic:Crop | 1.29 | 0.30 | | Organic:Crop | 5.83 | p<0.01 |
| | Organic | 37.68 | p<0.001 | | Organic | 54.00 | p<0.001 |
| WHC | Crop | 44.91 | p<0.001 | Na | Crop | 68.17 | p<0.001 |
| | Organic:Crop | 0.31 | 0.82 | | Organic:Crop | 17.44 | p<0.001 |
| | Organic | 33.32 | p<0.001 | | Organic | 19.97 | p<0.001 |
| $NH_4$-N | Crop | 16.91 | p<0.001 | CEC | Crop | 4.17 | 0.05 |
| | Organic:Crop | 41.25 | p<0.001 | | Organic:Crop | 2.71 | 0.07 |
| | Organic | 39.00 | p<0.001 | | Organic | 8.54 | p<0.001 |
| $NO_3$-N | Crop | 75.38 | p<0.001 | Macroaggregate | Crop | 4.46 | p<0.05 |
| | Organic:Crop | 16.83 | p<0.001 | | Organic:Crop | 1.49 | 0.25 |
| | Organic | 42.08 | p<0.001 | | Organic | 10.03 | p<0.001 |
| TN | Crop | 19.00 | p<0.001 | Microaggregate | Crop | 7.37 | p<0.05 |
| | Organic:Crop | 1.13 | 0.36 | | Organic:Crop | 1.89 | 0.16 |
| | Organic | 30.23 | p<0.001 | | Organic | 10.56 | p<0.001 |
| TC | Crop | 6.55 | p<0.05 | EG | Crop | 19.81 | p<0.001 |
| | Organic:Crop | 0.37 | 0.78 | | Organic:Crop | 10.24 | p<0.001 |
| | Organic | 7.19 | p<0.01 | | Organic | 34.24 | p<0.001 |
| CN | Crop | 0.16 | 0.70 | TG | Crop | 21.89 | p<0.001 |
| | Organic:Crop | 2.20 | 0.12 | | Organic:Crop | 0.13 | 0.94 |

**Table S2** Sum of relative abundance of the top 20 most abundant bacterial and fungal ASVs in cabbage cultivation system

| | Sum of relative abundance | |
|---|---|---|
| **Label** | **Bacteria** | **Fungi** |
| Con | 9.8±3.8 | 11.7±7.9 |
| Org A | 4.1±1.7 | 38.9±0.6 |
| Org B | 7.1±0.9 | 46.0±0.4 |
| Org C | 2.3±0.4 | 34.6±4.1 |

**Table S3** Sum of relative abundance of the top 20 most abundant bacterial and fungal ASVs in pepper cultivation system

| | Sum of relative abundance | |
|---|---|---|
| **Label** | **Bacteria** | **Fungi** |
| Con | 1.2±1.0 | 8.8±5.8 |
| Org A | 6.8±3.6 | 45.8±5.8 |
| Org B | 7.0±3.4 | 51.4±5.5 |
| Org C | 1.3±0.7 | 63.8±3.4 |

**Table S4** Spearman's rank correlation coefficients between microbial carbon use efficiency and bacterial amplicon sequence variants in cabbage cultivation systems. Correlation analysis between $^{13}$C-CUE values and relative abundance of the top 20 differentially abundant bacterial ASVs across organic farming duration treatments. Taxonomic classification is provided from phylum to genus level where available. Bold rho values indicate statistically significant correlations (P < 0.05)

| ASV | rho | Domain | Phylum | Class | Order | Family | Genus |
|---|---|---|---|---|---|---|---|
| ASV1 | **-0.83** | Bacteria | Proteobacteria | Gammaproteobacteria | Xanthomonadales | Xanthomonadaceae | - |
| ASV2 | **-0.80** | Bacteria | Actinobacteriota | Actinobacteria | Propionibacteriales | Nocardioidaceae | Nocardioides |
| ASV3 | **-0.83** | Bacteria | Planctomycetota | Planctomycetes | Planctomycetales | Schlesneriaceae | Schlesneria |
| ASV4 | **-0.86** | Bacteria | Proteobacteria | Alphaproteobacteria | Micropepsales | Micropepsaceae | uncultured |
| ASV5 | **-0.86** | Bacteria | Gemmatimonadota | Gemmatimonadetes | Gemmatimonadales | Gemmatimonadacea e | Gemmatimonas |
| ASV6 | -0.32 | Bacteria | Gemmatimonadota | Gemmatimonadetes | Gemmatimonadales | Gemmatimonadacea e | Gemmatimonas |
| ASV7 | -0.35 | Bacteria | Chloroflexi | KD4-96 | KD4-96 | KD4-96 | KD4-96 |
| ASV8 | -0.42 | Bacteria | Desulfobacterota | uncultured | uncultured | uncultured | uncultured |
| ASV9 | **-0.54** | Bacteria | Acidobacteriota | Acidobacteriae | Solibacterales | Solibacteraceae | Candidatus_Soliba cter |
| ASV10 | **-0.56** | Bacteria | Acidobacteriota | Vicinamibacteria | Vicinamibacterales | - | - |
| ASV11 | -0.06 | Bacteria | Patescibacteria | Saccharimonadia | Saccharimonadales | - | - |
| ASV12 | -0.07 | Bacteria | Proteobacteria | Gammaproteobacteria | Burkholderiales | Nitrosomonadaceae | MND1 |
| ASV13 | **-0.83** | Bacteria | Chloroflexi | Anaerolineae | SBR1031 | A4b | A4b |
| ASV14 | **0.53** | Bacteria | Chloroflexi | P2-11E | P2-11E | P2-11E | P2-11E |
| ASV15 | 0.34 | Bacteria | Proteobacteria | Gammaproteobacteria | Steroidobacterales | Steroidobacteraceae | - |
| ASV16 | -0.13 | Bacteria | Others | Others | Others | Others | Others |
| ASV17 | **0.57** | Bacteria | Bacteroidota | Bacteroidia | Chitinophagales | Saprospiraceae | uncultured |
| ASV18 | **0.56** | Bacteria | Bacteroidota | Bacteroidia | Chitinophagales | Saprospiraceae | uncultured |
| ASV19 | **0.69** | Bacteria | Bacteroidota | Bacteroidia | Chitinophagales | Saprospiraceae | uncultured |
| ASV20 | -0.40 | Bacteria | Planctomycetota | Phycisphaerae | Tepidisphaerales | WD2101_soil_group | WD2101_soil_gro up |

**Table S5** Spearman's rank correlation coefficients between microbial carbon use efficiency and fungal amplicon sequence variants in cabbage cultivation systems. Correlation analysis between $^{13}$C-CUE values and relative abundance of the top 20 differentially abundant fungal ASVs across organic farming duration treatments. Taxonomic classification is provided from phylum to genus level where available. Bold rho values indicate statistically significant correlations ($P < 0.05$)

| ASV | rho | Kingdom | Phylum | Class | Order | Family | Genus |
|---|---|---|---|---|---|---|---|
| ASV1 | **0.67** | Fungi | Mortierellomycota | Mortierellomycetes | Mortierellales | Mortierellaceae | - |
| ASV2 | 0.06 | Fungi | Ascomycota | Pezizomycetes | Pezizales | Ascobolaceae | Ascobolus |
| ASV3 | **0.68** | Fungi | Mortierellomycota | Mortierellomycetes | Mortierellales | Mortierellaceae | - |
| ASV4 | **0.66** | Fungi | Basidiomycota | Tremellomycetes | Filobasidiales | Piskurozymaceae | Solicoccozyma |
| ASV5 | -0.16 | Fungi | Basidiomycota | - | - | - | - |
| ASV6 | 0.08 | Fungi | Ascomycota | Pezizomycetes | Pezizales | Ascobolaceae | Ascobolus |
| ASV7 | -0.40 | Fungi | - | - | - | - | - |
| ASV8 | -0.22 | Fungi | Mortierellomycota | Mortierellomycetes | Mortierellales | Mortierellaceae | - |
| ASV9 | 0.53 | Fungi | Ascomycota | Leotiomycetes | Thelebolales | Thelebolales_fam_Incertae_sedis | unidentified |
| ASV10 | 0.05 | Fungi | Basidiomycota | Tremellomycetes | Cystofilobasidiales | Mrakiaceae | Tausonia |
| ASV11 | **0.58** | Fungi | Mucoromycota | Mucoromycetes | Mucorales | - | - |
| ASV12 | 0.41 | Fungi | Ascomycota | - | - | - | - |
| ASV13 | **0.71** | Fungi | Mortierellomycota | Mortierellomycetes | Mortierellales | Mortierellaceae | - |
| ASV14 | **0.59** | Fungi | Ascomycota | - | - | - | - |
| ASV15 | **0.58** | Fungi | Basidiomycota | Tremellomycetes | Cystofilobasidiales | Mrakiaceae | Tausonia |
| ASV16 | -0.23 | Fungi | Ascomycota | - | - | - | - |
| ASV17 | 0.08 | Fungi | Basidiomycota | Tremellomycetes | Filobasidiales | Piskurozymaceae | Solicoccozyma |
| ASV18 | 0.05 | Fungi | - | - | - | - | - |
| ASV19 | **0.85** | Fungi | Ascomycota | - | - | - | - |
| ASV20 | **0.80** | Fungi | Basidiomycota | - | - | - | - |

**Table S6** Spearman's rank correlation coefficients between microbial carbon use efficiency and fungal amplicon sequence variants in pepper cultivation systems. Correlation analysis between $^{13}$C-CUE values and relative abundance of the top 20 differentially abundant fungal ASVs across organic farming duration treatments. Taxonomic classification is provided from phylum to genus level where available. Bold rho values indicate statistically significant correlations (P < 0.05)

| ASV | rho | Kingdom | Phylum | Class | Order | Family | Genus |
|---|---|---|---|---|---|---|---|
| ASV1 | -0.01 | Fungi | Mortierellomycota | Mortierellomycetes | Mortierellales | Mortierellaceae | - |
| ASV2 | **0.65** | Fungi | Mortierellomycota | Mortierellomycetes | Mortierellales | Mortierellaceae | - |
| ASV3 | -0.13 | Fungi | Mortierellomycota | Mortierellomycetes | Mortierellales | Mortierellaceae | - |
| ASV4 | **0.56** | Fungi | Ascomycota | - | - | - | - |
| ASV5 | **0.58** | Fungi | Ascomycota | - | - | - | - |
| ASV6 | **0.60** | Fungi | - | - | - | - | - |
| ASV7 | 0.33 | Fungi | Mortierellomycota | Mortierellomycetes | Mortierellales | Mortierellaceae | - |
| ASV8 | 0.52 | Fungi | - | - | - | - | - |
| ASV9 | **0.77** | Fungi | Mucoromycota | Mucoromycetes | Mucorales | - | - |
| ASV10 | 0.14 | Fungi | Mortierellomycota | Mortierellomycetes | Mortierellales | Mortierellaceae | - |
| ASV11 | **0.64** | Fungi | Mortierellomycota | Mortierellomycetes | Mortierellales | Mortierellaceae | - |
| ASV12 | **0.62** | Fungi | Ascomycota | - | - | - | - |
| ASV13 | **0.54** | Fungi | Basidiomycota | - | - | - | - |
| ASV14 | **0.66** | Fungi | Basidiomycota | - | - | - | - |
| ASV15 | 0.24 | Fungi | Ascomycota | Dothideomycetes | Pleosporales | - | - |
| ASV16 | **0.59** | Fungi | Mucoromycota | Mucoromycetes | Mucorales | Rhizopodaceae | Rhizopus |
| ASV17 | **-0.86** | Fungi | Basidiomycota | - | - | - | - |
| ASV18 | 0.35 | Fungi | Basidiomycota | Tremellomycetes | Cystofilobasidiales | Mrakiaceae | Tausonia |
| ASV19 | 0.31 | Fungi | Basidiomycota | Agaricomycetes | - | - | - |
| ASV20 | 0.38 | Fungi | Basidiomycota | - | - | - | - |

**Fig. S1** Effects of organic farming duration on soil organic matter, glomalin-related soil protein fractions, and aggregate distribution in pepper cultivation systems. (a) Soil organic matter (SOM) content, (b) easily extractable glomalin (EG), (c) total glomalin (TG), and (d) sand-free water-stable aggregate distribution showing the proportion of macroaggregates (>250 μm) and microaggregates (53-250 μm). Different lowercase letters indicate significant differences among treatments (Duncan test, $P < 0.05$). Error bars represent standard deviations

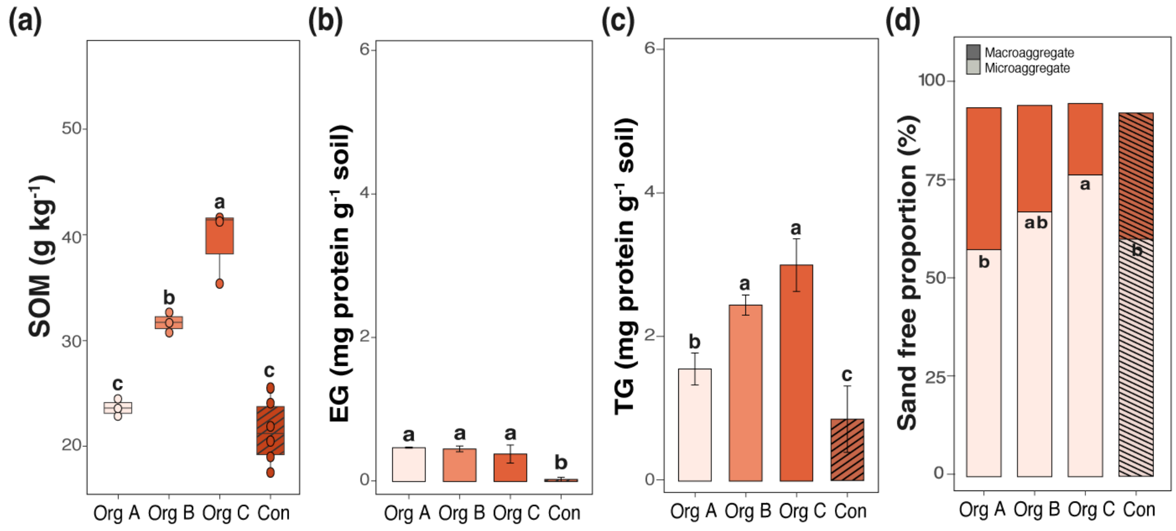

**Fig. S2** Differentially abundant microbial amplicon sequence variants (ASVs) in response to organic farming duration in pepper cultivation systems. Relative abundance of the top 20 most abundant (a) bacterial and (b) fungal amplicon sequence variants (ASVs) that showed significant responses to organic farming duration (one-way ANOVA or the Kruskal–Wallis test, $P < 0.05$) in pepper cultivation system. ASVs are classified at the phylum level in parentheses

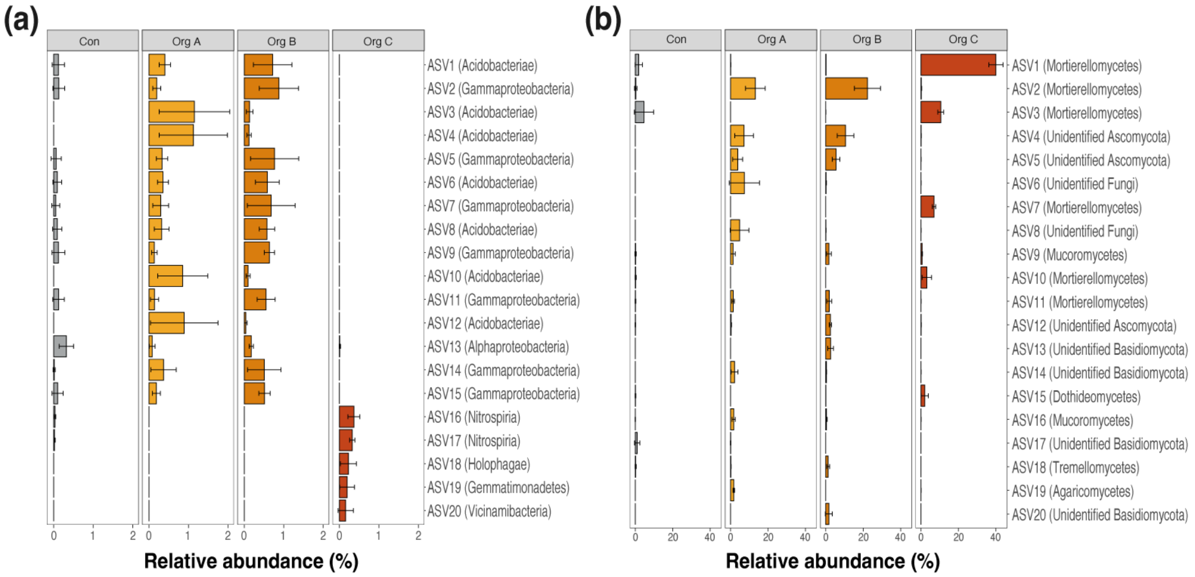

**Fig. S3** Microbial carbon use efficiency and extracellular enzyme activities in pepper cultivation systems under different organic farming durations. Microbial carbon use efficiency (CUE) measured using (a) $^{13}C$-glucose and (b) $^{18}O$-$H_2O$ labeling methods. Soil extracellular enzyme activities: (c) dehydrogenase activity (DHA), (d) N-acetyl-β-glucosaminidase (NAG), (e) β-glucosidase (BG), and (f) acid phosphatase (PP). Different lowercase letters indicate significant differences among treatments (Duncan test, P < 0.05)

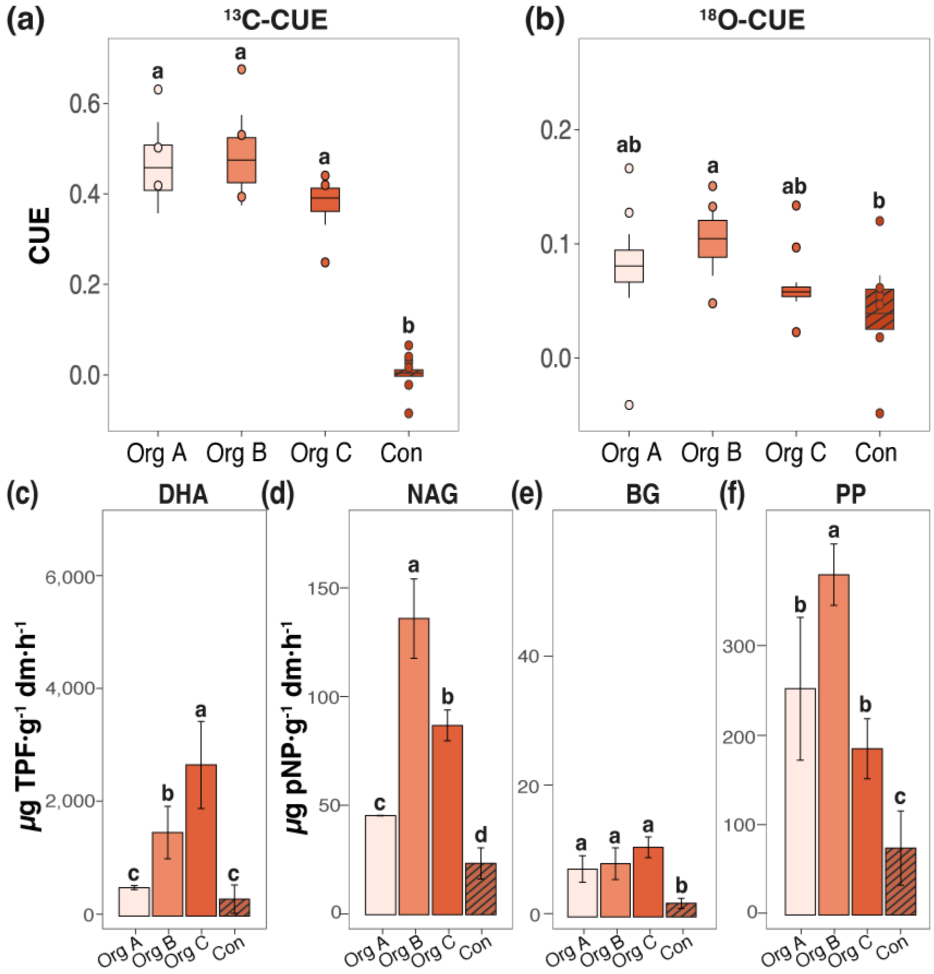

Supplement: Supplementary file 1 [file Data_Sheet_1.pdf]
